# Harvesting and Preparing *Drosophila* Embryos for Electrophysiological Recording and Other Procedures

**DOI:** 10.3791/1347

**Published:** 2009-05-20

**Authors:** David E. Featherstone, Kaiyun Chen, Kendal Broadie

**Affiliations:** Department of Biological Sciences, University of Illinois at Chicago; Department of Biological Sciences, Vanderbilt University

## Abstract

*Drosophila* is a premier genetic model for the study of both embryonic development and functional neuroscience. Traditionally, these fields are quite isolated from each other, with largely independent histories and scientific communities. However, the interface between these usually disparate fields is the developmental programs underlying acquisition of functional electrical signaling properties and differentiation of functional chemical synapses during the final phases of neural circuit formation. This interface is a critically important area for investigation. In *Drosophila*, these phases of functional development occur during a period of <8 hours (at 25°C) during the last third of embryogenesis. This late developmental period was long considered intractable to investigation owing to the deposition of a tough, impermeable epidermal cuticle. A breakthrough advance was the application of water-polymerizing surgical glue that can be locally applied to the cuticle to enable controlled dissection of late-stage embryos. With a dorsal longitudinal incision, the embryo can be laid flat, exposing the ventral nerve cord and body wall musculature to experimental investigation. This system has been heavily used to isolate and characterize genetic mutants that impair embryonic synapse formation, and thus reveal the molecular mechanisms governing the specification and differentiation of synapse connections and functional synaptic signaling properties.

**Figure:**
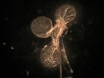


## Protocol

### Part 1: Equipment and Supplies

A good dissection microscope is required for embryo dissections; 40X magnification is suggested, with 25X eye-pieces to maximally increase magnification.
Fine forceps (number 5) are required for manual selection of embryos and devitellinization of embryos.
Equipment to make and modify fine glass needles is required.  Pulled-glass needles are required for the dissection. We prefer solid glass for dissection (last longer), but standard thick-walled glass tubing (outer diameter 1-1.5 mm) works as well. Some people use fine tungsten needles, electrolyzed to the desired sharpness in a bath of 1M NaOH using a 10+ Amp autotransformer battery. Sharpened tungsten dissection needles have the benefit that they are relatively resilient and long-lasting.  Hollow glass needles (formed similar to patch clamp electrodes) are attached to plastic tubing and used during dissection for suction and expulsion of saline, as well as the controlled delivery of glue. A variety of glass pullers are available for manufacturing glass needles. Computer-programmed pullers can be preset to pull a variety of shapes and sizes. The Brown-Flaming models (Sutter Instrument Co.) are widely favored. A compound microscope (20-40X objective) should be available to inspect and modify electrodes prior to use.
Two types of solutions are commonly used to bath the embryonic tissues during dissection: 1) “standard” (Jan and Jan, 1976a) or “modified standard” (Broadie, 2000) salines, based on solutions commonly used for recording in other invertebrate systems, and 2) “haemolymph-like” (HL) salines (Stewart et al 1994), a compromise between the standard saline and the ionic concentrations measured in the *Drosophila* haemolymph. It should be noted that none of these salines accurately mimic the chemical composition of haemolymph in vivo  (Broadie, 2000).  Standard saline contains (in mM): 135 NaCl, 5 KCl, 4 MgCl_2_, 1.8 CaCl_2_, 72 sucrose, 5 TES, pH 7.2. Experimenters may also want to consider commercially prepared insect saline (e.g. Schneider’s Insect Media), which is unsuitable for electrophysiological recording but most likely to protect the health of exposed tissues.

### Part 2: Embryo Staging and Dissection

For optimal egg collection, maintain young (<7 days), healthy male and female flies (20-40) on fresh laying pots for 2-3 days. Laying pots commonly consist of 100 ml plastic beakers (Tri-Pour) perforated to provide adequate air circulation, covering a 60 mm agar plate.  Agar plates contain apple or grape juice hardened with agar. Several recipes exist, but one example is as follows:  700 ml of water, 25-30 grams agar, 300 ml of juice concentrate (grape or apple), 0.5g methyl paraben (p-hydroxymethylbenzoate), and 30g sugar.  Autoclave the water and agar, and separately boil the p-hydroxymethylbenzoate with the sugar and juice.  Mix the agar and juice solutions and quickly pour into 60 mm plates, removing bubbles.
Prior to egg collection, flies are fed with yeast paste (7 grams baker’s yeast in 9 ml of water stored at 4°C) on fresh plates, changed at least twice a day for at least two days.  By day 3, a good pot should produce 100-200 eggs per hour.  Better egg laying is observed on a pot maintained on its side with the agar in the plate scratched.  Many embryos will be laid in or next to the scratches.
To collect large numbers of embryos, gently transfer the embryos from the agar plate into a basket using a paintbrush. A basket can be made with a 15 or 50 ml centrifuge tube.  Cut off the bottom leaving enough surface area to trap a screen (70 µm mesh) between the lid and the top of the tube.  Rinse the embryos with dH_2_0 and put into a Petri dish of fresh 50% to 100% bleach to remove the outer chorion.  Alternatively, eggs can be collected individually using fine forceps, and dechorionated by placing them manually into a drop of bleach.  De-chorionation can take from 30 seconds to 2 minutes (fresh bleach is much faster), and should be monitored under the dissection microscope.  Removal of the chorion exposes the shiny, transparent vitelline membrane.  Dechorionated eggs should be briefly rinsed, or placed in saline, as bleach rapidly destroys devitellinized tissues.
It is critical to carefully stage embryos to the desired developmental age. The defined *Drosophila* embryonic stages (1-17; Campos-Ortega and Hartenstein, 1985) are not useful, as all functional neuromuscular development occurs in late stage 16 or stage 17. Thus, embryos are staged by developmental hours at 25°C in a humidified incubator, in hours after fertilization or, more commonly, after egg laying (AEL). Under these conditions, embryogenesis lasts 21 +/- 1 hr at 25°C.
Eggs from a timed egg-lay (1-2 hrs) that are appropriately aged are next viewed for morphological criteria. After extensive washing in dH_2_O, the dechorionated eggs are placed in a plastic culture dish (under dH_2_O) for viewing. Correct staging can be confirmed with morphological criteria using reflected light with a dissection microscope (Campos-Ortega and Hartenstein, 1985). Mature late-stage embryos (22-24h AEL) on the verge of hatching are generally recognized by segmentation of the cuticle and inflated trachea.  Embryos can also be genotyped using GFP balancers and a fluorescence dissection microscope with appropriate filters.
For dissection, dechorionated and developmentally staged embryos are attached (dorsal side up) on a coverslip under a drop of saline (see step 1.5). Embryos are removed from the vitelline membrane by puncturing the membrane near the anterior or posterior end with a glass micropipette or tungsten needle, and then gently freeing the embryo from the membrane. In preparation for dissection, the embryo should be positioned on the coverslip with the dorsal surface up (Dorsal side up dissection will expose the ventral nervous system and neuromusculature for experiments.  However, the embryo can obviously be positioned in other ways to facilitate access to other tissues. )
Early stage embryos (<16 hrs AEL) attach directly to clean glass or glass coated with polylysine (poly-L-lysine hydrobromide; Broadie and Bate, 1993a, b). Older embryos (>16 hrs AEL), following cuticle formation, must be glued, ideally to Sylgard-coated (Dow Corning) coverslips. A water-polymerizing cyanoacrylate surgical or veterinary glue is used (Broadie and Bate, 1993c, d). The glue is delivered through a small glass electrode pipette (10-20 micrometer inner diameter tip) attached to a rubber tube with the glue flow controlled by mouth pressure. Care must be taken during glue delivery, as the glue polymerizes quickly upon contacting the saline. Note also that gluing cannot be performed in saline with no or low divalent ion concentrations, as the glue requires divalent ions to polymerize.  Small amounts of glue are first used to firmly attach the head and tail.
Once the ends of the embryo are glued to the coverslip, an incision is made along the dorsal midline with a glass electrode or sharpened tungsten needle. This is made easier by gently perforating the line of incision prior to making the final cut. The sides of the incision are then attached to the coverslip with more glue, to gently spread the embryo flat. The internal organs including the gut, fat bodies and, optionally, trachea are then removed by suction using a glass pipette attached to rubber tubing (Broadie and Bate 1993a-c). The embryo should now be attached flat to the coverslip, epidermis down with the ventral central nervous system, peripheral nerves, and somatic musculature exposed for experimentation.

## Discussion

Precise staging of embryos is critical due to the rapid maturation of functional properties over the time course of just several hours. Several issues complicate this staging. First, most researchers use timed egg lays to stage embryos, but the egg laying time can vary enormously from animal to animal in different conditions (Broadie et al. 1992). In particular, females on a limited diet tend to retain fertilized eggs for prolonged periods prior to laying. It is therefore critical to “clear” females by feeding on a rich yeast diet for at least 2 days prior to collecting eggs (Broadie et al. 1992). Moreover, older females also retain eggs for a longer period prior to laying. An older female may routinely lay eggs only a couple hours prior to their hatching. It is therefore important to use young females (<7 days) for the most consistent laying times (Broadie et al. 1992). Second, during late embryo stages, it is difficult to stage embryos solely by morphological criteria. Most clear staging features are complete by <16 hrs AEL (Campos-Ortega and Hartenstein, 1985), but most functional development occurs >16 hrs AEL. Late developmental features (e.g. tracheal air-filling, tanning of cuticle) are few and tend to be less temporally restricted. For these reasons, we recommend a combinatorial approach: collect eggs from 1-2 hr timed lays, stage to well-defined early morphological events of <30 minutes in duration (e.g. gastrulation, dorsal closure, 3-part gut; Campos-Ortega and Hartenstein, 1985) and then incubate to the desired age in a well-controlled 25°C incubator.

The manual dissection and glue application require fine motor skills under a microscope that few people initially possess. These skills must be developed over time. Experimenters should be willing to commit a minimum of several weeks of daily dedicated practice before expecting a high frequency of success. Preparations suitable for microscopic examination of the CNS and some ventral neuromuscular junctions should be achievable relatively quickly, while healthy preparations suitable for electrophysiology will typically require more effort.
